# Anti‐IFN‐α/‐ω neutralizing antibodies from COVID‐19 patients correlate with downregulation of IFN response and laboratory biomarkers of disease severity

**DOI:** 10.1002/eji.202249824

**Published:** 2022-04-28

**Authors:** Federica Frasca, Mirko Scordio, Letizia Santinelli, Lucia Gabriele, Orietta Gandini, Anna Criniti, Alessandra Pierangeli, Antonio Angeloni, Claudio M. Mastroianni, Gabriella d'Ettorre, Raphael P. Viscidi, Guido Antonelli, Carolina Scagnolari

**Affiliations:** ^1^ Laboratory of Microbiology and Virology Department of Molecular Medicine Sapienza University of Rome Rome Italy; ^2^ Department of Oncology and Molecular Medicine Istituto Superiore di Sanità Rome Italy; ^3^ Department of Molecular Medicine Sapienza University of Rome Rome Italy; ^4^ Department of Experimental Medicine Policlinico Umberto I Sapienza University of Rome Rome Italy; ^5^ Department of Public Health and Infectious Diseases Policlinico Umberto I Sapienza University of Rome Rome Italy; ^6^ Department of Pediatrics Johns Hopkins University School of Medicine Baltimore Maryland USA

**Keywords:** COVID‐19, Interferon, ISG, neutralizing antibodies, binding antibodies, autoantibodies, SARS‐CoV‐2

## Abstract

A significant number of COVID‐19 patients were shown to have neutralizing antibodies (NAB) against IFN; however, NAB specificity, fluctuation over time, associations with biochemical and hematological parameters, and IFN gene expression are not well characterized.

Binding antibodies (BAB) to IFN‐α/‐β were screened in COVID‐19 patients’ serum. All BAB positive sera, and a subset of respiratory samples, were tested for NAB against IFN‐α/‐β/‐ω, using an antiviral bioassay. Transcript levels of IFN‐α/‐β/‐ω and IFN‐stimulated genes (ISGs) were quantified.

Anti‐IFN‐I BAB were found in 61 out of 360 (17%) of patients. Among BAB positive sera, 21.3% had a high NAB titer against IFN‐α. A total of 69.2% of anti‐IFN‐α NAB sera displayed cross‐reactivity to IFN‐ω. Anti‐IFN‐I NAB persisted in all patients. NAB to IFN‐α were also detected in 3 out of 17 (17.6%) of respiratory samples. Anti‐IFN‐I NAB were higher in males (*p* = 0.0017), patients admitted to the ICU (*p* < 0.0001), and patients with a fatal outcome (*p* < 0.0001). NAB were associated with higher levels of CRP, LDH, d‐Dimer, and higher counts of hematological parameters. ISG‐mRNAs were reduced in patients with persistently NAB titer.

NAB are detected in a significant proportion of severe COVID‐19. NAB positive patients presented a defective IFN response and increased levels of laboratory biomarkers of disease severity.

## Graphical abstract

Autoantibodies capable of neutralizing multiple IFN‐α subtypes and IFN‐ω can be found in severe COVID‐19 patients, preferentially males. Anti‐IFN‐I neutralizing antibodies (NAB) positive patients present a defective IFN response and have raised levels of biochemical and hematological parameters predictive of severe COVID‐19.

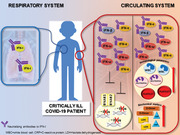

## Introduction

The phenotypic heterogeneity of SARS‐CoV‐2 infection spans asymptomatic to severe disease and death. Although the causes of COVID‐19 remain to be established, increasing evidence demonstrates that defects in type I interferon (IFN‐I) responsiveness may represent an important pathogenetic mechanism [1, 2, 3, 4]. Recent studies have shown that SARS‐CoV‐2 triggers a low and delayed IFN response in some critically ill patients with the response varying based on viral load, age, and disease severity [2, 4, 5]. The mechanisms of altered production of IFN‐I are unknown, but several SARS‐CoV‐2 viral proteins have been shown to strongly suppress IFN induction [6]; in some patients the defect is explained by inborn genetic defects of IFN‐I immunity [7]; and, in other patients by the presence of neutralizing antibodies (NAB) directed against IFN‐I [8]. These antibodies are known as natural autoantibodies (auto‐Abs) to IFN to distinguish them from those induced by IFN‐α or ‐β treatment [9]. The production of anti‐IFN‐I NAB is associated with an impaired IFN‐I response and severe COVID‐19 pneumonia in approximately 10% of patients [8]. Detection of anti‐IFN‐I NAB in SARS‐CoV‐2 positive patients has been proposed as a predictive marker for high‐risk populations and adverse outcomes of COVID‐19.

We investigated the prevalence of anti‐IFN‐I auto‐Abs in a cohort of SARS‐CoV‐2 positive patients from a single hospital in Rome, Italy, and examined their specificity, fluctuation over time, biological significance, and impact on biochemical and hematological parameters of COVID‐19 severity.

## Results

### Natural autoantibodies against IFN‐I in serum samples of COVID‐19 patients

**Figure 1:**
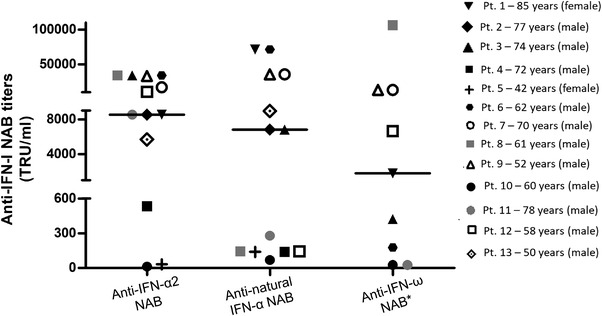
**Broad anti‐IFN‐I‐neutralizing antibody titers in COVID‐19 patients**. Individual anti‐IFN‐α2, anti‐natural IFN‐α, and anti‐IFN‐ω NAB titers detected in serum samples from COVID‐19 patients by antiviral bioassay (n = 13). Each individual is represented by a distinct symbol, age (years), and gender. Median values are represented with a black horizontal line. * 9 out of 13 NAB positive patients had anti‐IFN‐ω NAB.

Binding antibodies (BAB) against IFN detectable by ELISA comprise both NAB and non‐NAB. We first measured BAB against IFN‐α subtypes and IFN‐β in serum samples collected from 360 hospitalized COVID‐19 patients. The median age of the patients was 63 years (range: 99–24); 69.2% (249/360) were male; 16.7% (60/360) were admitted to the intensive care unit (ICU); and 13.6% (49/360) had a fatal outcome. The overall prevalence of anti‐IFN‐α BAB was 7.5% (27/360) and that of antibodies to IFN‐β was 10.3% (37/360) (Table 1). Three COVID‐19 patients had BAB against both IFN‐α and ‐β. NAB bioassays were performed on serum samples with anti‐IFN‐α/β BAB positivity (n = 61, Table 1). NAB against IFN‐α2 subtype were detectable more frequently than NAB against IFN‐β [48.1% (13/27) versus 2.7% (1/37), respectively, *p* < 0.0001, using Yates Chi square test). The single serum sample positive for anti‐IFN‐β NAB was also positive for anti‐IFN‐α2 subtype auto‐Abs. The range of NAB levels against IFN‐α2 subtype was very broad (34133–13 tenfold reduction unit [TRU/mL]) (Fig. 1). All serum samples with NAB against IFN‐α2 subtype were able to neutralize the IFN‐α subtypes contained in the natural IFN‐α preparation, IFN‐αn1 (Fig. 1). Levels of NAB to IFN‐α2 subtype and to the natural IFN‐α preparation were not correlated (*p* = 0.07; *r* = 0.50, using Spearman's rho coefficient). NAB to IFN‐α2 were higher in males than females (*p* = 0.0017, using Yates Chi square test), in COVID‐19 patients admitted to the ICU (*p* < 0.0001, using Yates Chi square test), and in those who had a fatal outcome of infection (*p* < 0.0001, using Yates Chi square test) (Table 1). No significant differences were observed with respect to patients’ age (*p* = 0.1168, using Yates Chi square test) (Table 1).

**Table 1:** Frequency of binding (BAB) and neutralizing (NAB) antibodies to IFN‐I (IFN‐α and IFN‐β), demographic and clinical parameters of COVID‐19 patients

COVID‐19 patients	Anti‐IFN‐α/β BAB negative	Anti‐IFN‐α BAB positive	Anti‐IFN‐β BAB positive	Anti‐IFN‐α NAB positive^^^	Anti‐IFN‐β NAB positive
Total n = 360	299/360 (83)	27/360 (7.5)	37/360 (10.3)	13/360 (3.6)	1/360 (0.3)
Gender					
Male (n = 249)	202/299 (67.6)	^*^24/27 (88.9)	^**^24/37 (64.9)	^***^11/13 (84.6)	0/1 (0)
Female (n = 111)	97/299 (32.4)	3/27 (11.1)	13/37 (35.1)	2/13 (15.4)	1/1 (100)
Age					
^Δ^ <60 years (n = 156)	132/299 (44.1)	11/27 (40.7)	14/37 (37.8)	4/13 (30.8)	1/1 (100)
≥60 years (n = 201)	165/299 (55.2)	15/27 (55.6)	23/37 (62.2)	9/13 (69.2)	0/1
ICU admission	42/299 (14)	4/14 (28.6)	4/35 (11.4)	^°^10/13 (76.9)	^†^1/1 (10
Death rate	32/299 (10.7)	3/14 (21.4)	4/35 (11.4)	^°^10/13 (76.9)	^■^1/1 (100)

Data are expressed as total number (%) of COVID‐19 patients negative to anti‐IFN‐I BAB or positive to anti‐IFN‐α or ‐β BAB and NAB.

^^^Anti‐IFN‐α NAB were detected against IFN‐α2 subtype and multiple IFN‐α subtypes contained in the natural IFN‐α preparation (IFN‐αn1, Wellferon Glaxo Wellcome, Beckenham, United Kingdom). Statistical analysis was performed using Yates Chi‐square.

^*^ ***p* < 0.0001** for anti‐IFN‐α BAB of male patients versus anti‐IFN‐α BAB of female patients.

^**^ ***p* = 0.02** for anti‐IFN‐β BAB of male patients versus anti‐IFN‐β BAB of female patients.

^***^ ***p* = 0.0017** for anti‐IFN‐α NAB of male patients versus anti‐IFN‐α NAB of female patients.

^°^ ***p *< 0.0001** for intensive care unit (ICU) admission and death rate of anti‐IFN‐α NAB positive patients versus anti‐IFN‐α BAB positive and negative ones.

^†^ ***p* = 0.0424** for ICU of anti‐IFN‐β NAB positive patients versus anti‐IFN‐β BAB positive and negative ones.

^■^ ***p* = 0.0175** for death rate of anti‐IFN‐β NAB positive patients versus anti‐IFN‐β BAB positive and negative ones. ^Δ^Data are available for 357 out of 360 COVID‐19 patients.

### Specificity of NAB against IFN‐α and IFN‐ω in COVID‐19 patients

COVID‐19 patients might have developed a broad spectrum of NAB with specificity against different IFN‐I molecules. We found that 69.2% (9/13) of sera containing anti‐IFN‐I NAB were able to neutralize IFN‐ω. While 42.9% (3/7) of sera with low or intermediate titers of anti‐IFN‐α2 NAB (<10.000 TRU/mL) had auto‐Abs against IFN‐ω, 100% (6/6) of sera with high titers of anti‐IFN‐α2 NAB (≥10.000 TRU/mL) were able to neutralize IFN‐ω (Supporting information Table S1). Anti‐IFN‐ω NAB were associated with male sex (*p* = 0.0034, using Fisher's exact test, Supporting information Table S1), admission to the ICU and fatal outcome (*p* < 0.0001 for both parameters, using Yates Chi square test, Supporting information Table S1).

### Anti‐IFN‐I NAB associate with laboratory biomarkers predictive for COVID‐19 outcome

**Figure 2:**
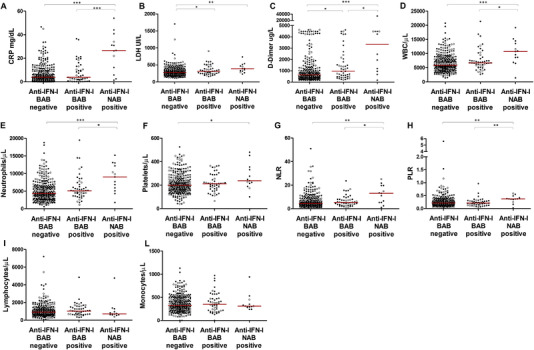
Anti‐IFN‐I NAB were associated **with laboratory biomarkers predictive for COVID‐19 outcome**. Biochemical and hematological parameters levels in SARS‐CoV‐2‐infected patients, stratified by autoantibody status as anti‐IFN‐I BAB negative patients (total n = 299), anti‐IFN‐I BAB positive patients without NAB to IFN‐α subtypes, IFN‐β, and IFN‐ω (total n = 48), and anti‐IFN‐I NAB positive patients (total n = 13). CRP, C‐reactive protein (Panel A); LDH, lactate dehydrogenase (Panel B); d‐Dimer (Panel C); WBC, white blood cells (Panel D); neutrophils (Panel E); platelets (Panel F); NLR, neutrophils to lymphocytes ratio (Panel G); PLR, platelets to lymphocytes ratio (Panel H); lymphocytes (Panel I); monocytes (Panel L). Median values of biochemical and hematological parameters are reported, for each group of study, with a red horizontal line. Female patients are represented with open circle symbols while male patients with close circle symbols. *p < 0.05; **p < 0.01; ***p ≤ 0.001. Values of biochemical and hematological parameters were compared by Mann–Whitney test.

Detection of NAB against IFN‐I has been associated with a poor outcome of COVID‐19 [10, 11, 12]. We compared levels of laboratory biomarkers (CRP [C‐reactive protein], lactate dehydrogenase [LDH], d‐Dimer, total white blood cells [WBC], neutrophils, platelets, neutrophils to lymphocytes ratio [NLR], platelets to lymphocytes ratio [PLR], lymphocytes and monocytes) associated with high risks for severe COVID‐19 [13, 14, 15], between COVID‐19 patients with anti‐IFN‐I NAB (n = 13), and those negative for both BAB and NAB to IFN‐I (n = 299). Levels of CRP (Panel A), LDH (Panel B), and d‐Dimer (Panel C) were higher in patients with anti‐IFN‐I NAB compared to those negative for anti‐IFN‐I BAB (Fig. 2, *p* < 0.01 for all the determinations, using Mann–Whitney test). With respect to hematological parameters, we found that patients with anti‐IFN‐I NAB had increased counts of total WBC (Panel D), neutrophils (Panel E), platelets (Panel F), NLR (Panel G), and PLR (Panel H) compared to patients negative for anti‐IFN‐I BAB (Fig. 2, *p* < 0.05 for all the determinations, using Mann‐Whitney test). A nonsignificant trend was observed in patients with anti‐IFN‐I NAB compared to those negative for anti‐IFN‐I BAB for a reduction in lymphocytes (Panel I) and monocytes (Panel L) (Fig. 2). Similar results were found for biochemical and hematological parameters when the analysis was restricted to males with anti‐IFN‐I NAB antibodies (Fig. 2). Increased levels of CRP, LDH, d‐Dimer, total WBC, neutrophils, NLR, and PLR were also observed among anti‐IFN‐ω NAB positive patients (*p* < 0.05 for all the determinations, using Mann‐Whitney test, Supporting information Fig. S1, Panels A‐D, E, G, and H) and anti‐IFN‐I BAB negative ones. COVID‐19 patients with only BAB to IFN‐α/β (n = 48) did not exhibit significantly enhanced levels in laboratory parameters compared to those without anti‐IFN‐I antibodies, with the only exception of LDH (Panel B) and d‐Dimer (Panel C) (Fig. 2, *p* < 0.05 for all the determinations, using Mann‐Whitney test).

### Circulating anti‐IFN‐I NAB correlate with inhibition of IFN gene expression in COVID‐19 patients

**Figure 3:**
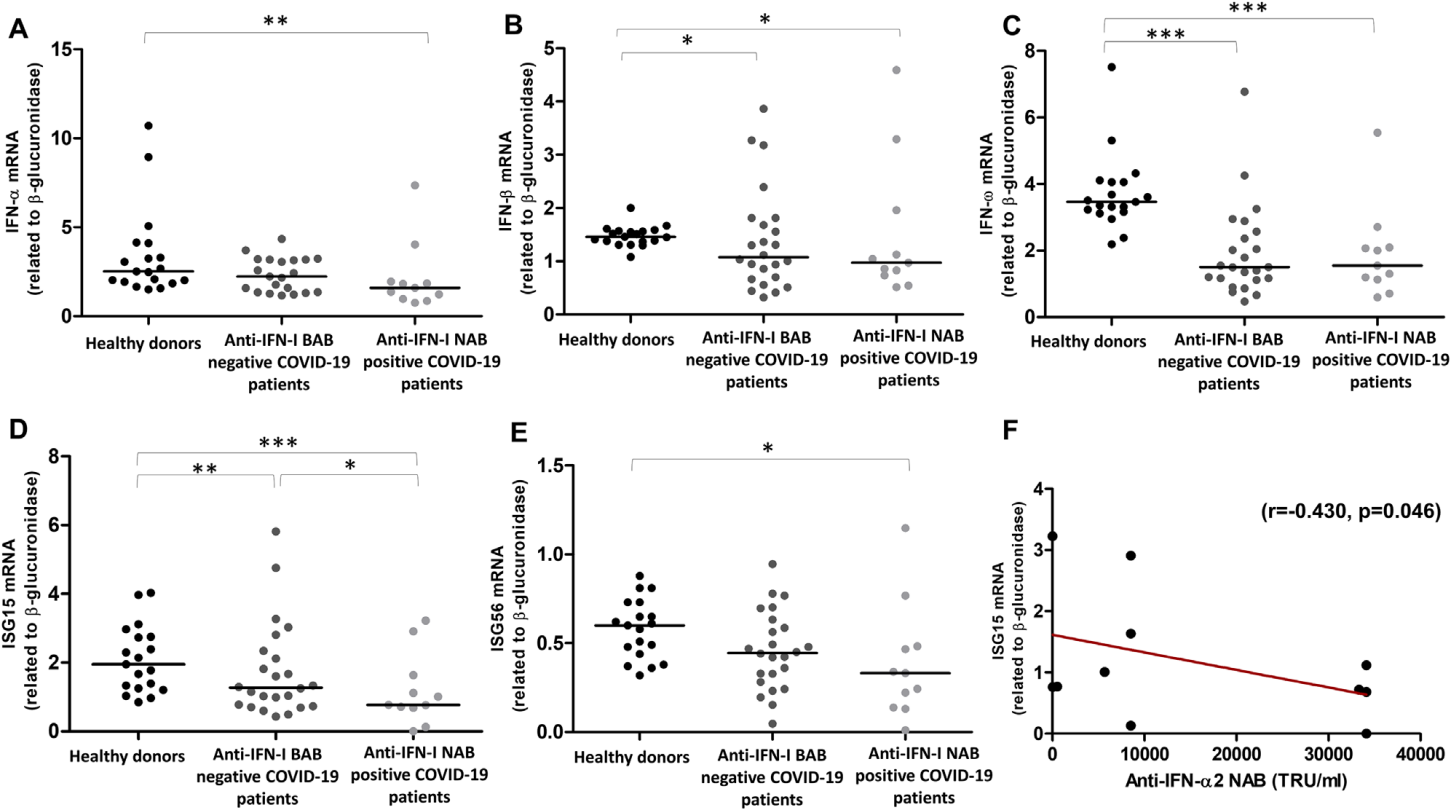
**Expression levels of IFN‐I and IFN‐stimulated genes in anti‐IFN‐I NAB positive COVID‐19 patients**. Panels A‐E represent expression levels of genes, encoding IFN‐α (A), IFN‐β (B), IFN‐ω (C), ISG15 (D), and ISG56 (E), measured by real time PCR, in PBMC collected from healthy donors (n = 19), anti‐IFN‐I BAB negative COVID‐19 patients (n = 24), and those who developed anti‐IFN‐I NAB (n = 11). Gene expression analysis was available for 11 out of 13 anti‐IFN‐I NAB positive patients. Panel F indicates the correlation between ISG15 mRNA expression levels and anti‐IFN‐α2 NAB titer. Statistical analysis of transcript levels of IFN genes related to β‐glucuronidase (2^−Δ^ *^Ct^* method), was carried out using Mann–Whitney test (Panels A‐E). Correlation was assessed using Spearman's *ρ* coefficient (*p* < 0.05, Panel F). Median values of gene expression levels (Panels A–E) are reported, for each group of study, with a black horizontal line. ^∗^ *p* < 0.05; ^∗∗^ *p* < 0.01; ^∗∗∗^ *p* <0.001.

Reduction and/or abrogation of the endogenous‐induced IFN response has been associated with COVID‐19 severity and in particular with high titers of anti‐IFN‐I NAB [10, 11, 16]. We performed expression analysis of IFN‐I genes (IFN‐α, ‐β, and ‐ω) and two IFN‐stimulated genes (ISGs), ISG15 and ISG56, in COVID‐19 patients who were positive for anti‐IFN‐I NAB, in gender and age‐matched control patients negative for BAB to anti‐IFN‐I (n = 24) and gender and age‐matched healthy individuals (n = 19). We found that, compared to healthy individuals, COVID‐19 patients had a reduced gene expression of IFN‐β, IFN‐ω, and ISG15 in blood cells, independently of the presence of anti‐IFN‐I NAB (Fig. 3, Panels B, C, and D, *p* < 0.05 for all genes, using Mann‐Whitney test). Moreover, anti‐IFN‐I NAB positive patients had a trend toward lower expression of IFN‐I genes (IFN‐α, ‐β, ‐ω, and ISG56) compared to patients negative for anti‐IFN‐I BAB (Fig. 3, Panels A‐C and E). The level of ISG15 mRNA was reduced in SARS‐CoV‐2‐infected patients positive for anti‐IFN‐I NAB compared to those without anti‐IFN‐I BAB (*p* < 0.05, Fig. 3, Panel D, using Mann‐Whitney test). Similar associations were seen when the analysis was restricted to anti‐IFN‐ω NAB positive patients, with statistically significant differences for IFN‐ω and ISG15 (*p* < 0.01 for both genes using Mann‐Whitney test, Supporting information Fig. S2, Panels C and D) and a nonsignificant trend for the other IFN genes, perhaps due to the limited number of NAB positive samples to IFN‐ ω (Supporting information Fig. S2, Panels A, B, and E). Additionally, a negative correlation was found between NAB titer against IFN‐α2 subtype and ISG15 transcript levels (*r* = −0.430, *p* = 0.046, Fig. 3, Panel F, using Spearman's rho coefficient).

### Persistence of anti‐IFN‐I NAB and inhibition of IFN genes in COVID‐19 patients

**Figure 4:**
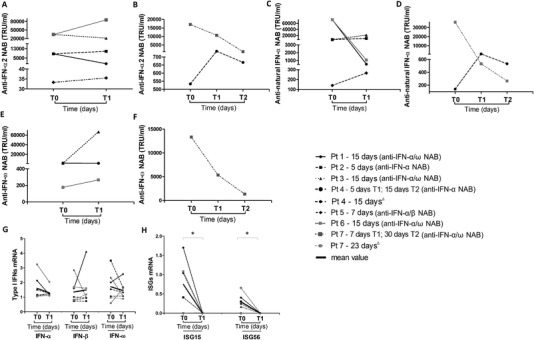
**Persistence of anti‐IFN‐I NAB and inhibition of IFN‐related genes in COVID‐19 patients**. Temporal changes in anti‐IFN‐α2, anti‐natural IFN‐α, and anti‐IFN‐ω NAB titers and mRNA levels of IFN‐I (IFN‐α, IFN‐β, IFN‐ω), ISG15 and ISG56, measured by antiviral bioassay and RT‐real time PCR, respectively, in COVID‐19 patients (n = 7) at different time points after hospitalization. Each patient is represented by a distinct line. The interval time expressed in days elapsed between T0 (time of hospitalization) and T1 (Panels A, C, and E) and between T0, T1, and T2 (Panels B, D, and F) is indicated for each patient near the corresponding line together with the NAB status. In Panel H, levels of ISGs measured at T1 were undetectable (*Ct* values < 45). For the statistical analysis levels of ISG15 and ISG56 related to β‐glucuronidase were calculated using 2^−Δ^ *^Ct^* method assuming the *Ct* value as 45 Ct. Statistical analysis were performed using Wilcoxon test. ^∗^ *p* = 0.0002. ^Δ^ Longitudinal observation of IFN‐I and ISGs levels of Pt 4 and Pt 7 are reported in Panels G and H.

Few studies have performed longitudinal measurements of auto‐Abs to IFN‐I during SARS‐CoV‐2 infection [11]. We tested a subgroup of COVID‐19 patients positive for anti‐IFN‐I NAB (n = 7) for auto‐Abs at different time points after hospitalization (median interval of 15 days [interquartile range: 7–15]). All patients exhibited persistent NAB positivity to IFN‐α2 subtype and against the IFN‐α subtypes contained in the natural IFN‐α preparation. Anti‐IFN‐α NAB titers showed fluctuation over time but remained elevated (median/range at T1 and T2: IFN‐α2 subtype [10667/85333‐35 TRU/mL and 867/1067‐667 TRU/mL]; natural IFN‐α preparation [1067/21333‐267 TRU/mL and 400/533‐267 TRU/mL]) in COVID‐19 patients (Fig. 4, Panels A‐D). Longitudinal observations in four out of the seven COVID‐19 patients who were positive for anti‐IFN‐ω auto‐Abs showed persistence of high titers of anti‐IFN‐ω NAB at T1 (median/range: 3333/66 667–267 TRU/mL) and T2 (130 TRU/mL), (Fig. 4, Panels E and F), respectively.

Analysis of transcript levels of IFN‐α, ‐β, and ‐ω showed fluctuations over time in COVID‐19 patients, but median levels did not change significantly (Fig. 4, Panel G). By contrast, mRNA levels of ISG15 and ISG56 were reduced to an undetectable level (cycle threshold/*Ct* values less than 45) in patients who had persistently high titers of anti‐IFN‐α or anti‐IFN‐ω NAB (Fig. 4, Panel H, *p* = 0.0002 for both genes, using Wilcoxon test).

### Correlation of anti‐IFN‐I NAB and IFN response in respiratory samples of COVID‐19 patients

We measured NAB to IFN‐α, ‐β, and ‐ω in the supernatants of respiratory samples (nasopharyngeal swabs [NPS] (n = 6) and bronchoalveolar lavage [BAL] [n = 11]) collected from 17 COVID‐19 patients for whom paired serum samples were available (Supporting information Table S2). NAB against IFN‐α2 subtype were detected in three BAL samples and two out of three BAL samples also showed NAB positivity to natural IFN‐α preparation (Supporting information Table S2). Titers of NAB against IFN‐α2 subtype and natural IFN‐α preparation were lower (IFN‐α2, 20–10 TRU/mL; natural IFN‐α preparation, 15–10 TRU/mL) compared to serum levels. Respiratory tract samples did not contain NAB against IFN‐β and IFN‐ω (Supporting information Table S2). We did not find differences in expression levels of IFN‐I genes, or ISG56 and ISG15 in respiratory samples comparing patients positive and negative for NAB against IFN‐α subtypes (Supporting information Fig. S3).

## Discussion

A highly impaired IFN‐I response is known to underlie severe COVID‐19 [1, 2, 3]. In a subset of COVID‐19 patients, the defect is explained by the presence of auto‐Abs against IFN‐I [8, 10, 11, 12]. We found that antibodies capable of binding to IFN‐α subtypes and/or IFN‐β can be detected in up to 17% of hospitalized COVID‐19 patients and about one of five of these serum samples contained auto‐Abs that neutralized IFN‐I. Our results confirm previous studies in COVID‐19 patients [8, 10]. NAB were preferentially found in those SARS‐CoV‐2‐infected patients who required ICU admission and had a fatal outcome of infection. In our population, these patients were preferentially males between the ages of 78 and 50 years. An association of male sex with anti‐IFN‐I NAB has been reported in some but not all previously published studies [8, 10, 11, 17, 18]. There was a significant correlation between NAB and elevated levels of CRP, LDH, and d‐Dimer, which are well‐established indicators of a worse COVID‐19 prognosis [19, 20]. NAB, but not BAB, were also associated with elevations in total WBC counts, neutrophils, platelets, PLR, and NLR values (Fig. 2), which are hematological abnormalities associated with severe COVID‐19 in previous studies [12]. Low titers of NAB to IFN‐α subtypes were detected in BAL samples collected from three COVID‐19 patients admitted to the ICU, in agreement with a previous study [16]. Moreover, we found that only one patient out of three with respiratory anti‐IFN‐α NAB had auto‐Abs in paired serum sample (33.3%, n = 1/3), supporting an earlier report showing COVID‐19 patients with serum anti‐IFN‐I NAB can have these antibodies in the respiratory tract [16]. In our study, NAB were detected only in BAL samples; this may be due to the small number of respiratory samples collected or to the quality of the nasopharyngeal swab samples.

We found that the prevalence of BAB to IFN‐I in COVID‐19 individuals was higher than that of NAB, consistent with reports of other cohorts of COVID‐19 patients [8, 10]. We found a significantly higher prevalence of neutralizing activity against IFN‐α (48% [13/27], Table 1) compared to IFN‐β (2.7% [1/37], Table 1) and the single patient out of 13 NAB positive patients with anti‐IFN‐β NAB also had anti‐IFN‐α NAB.

Our findings that anti‐IFN‐β NAB are rarely detected in COVID‐19 individuals is in agreement with Bastard et al., who found only 2 out of 19 COVID‐19 patients with anti‐IFN‐α auto‐Abs had anti‐IFN‐β antibodies [8]. The low prevalence of anti‐IFN‐β NAB in COVID‐19 patients might have pathological consequences since there are differences between IFN‐α and ‐β in IFN receptor binding affinity and biological activities [21, 22]. The low prevalence of anti‐IFN‐β NAB might allow for treatment with IFN‐β in severe COVID‐19 patients with anti‐IFN‐α NAB [12]. Indeed, it is known that when IFN‐α has been used to treat thrombocytosis, chronic hepatitis B and C, and certain types of cancer, NAB are associated with loss of clinical effectiveness [23, 24, 25, 26]. As the risk of developing severe and even potentially fatal COVID‐19 is high in patients with NAB, the optimal use of IFN‐I in COVID‐19 needs to be better defined since its exploration as an emergency treatment in various clinical trials [27] has excluded patients with demonstrable auto‐Abs to IFN‐α.

We found that IFN‐ω, which shares only approximately 60% amino acid homology to IFN‐α [28], was recognized by most of the sera which neutralized IFN‐α. These results confirm previous observations that IFN‐α and IFN‐ω, but not IFN‐β, were neutralized to a similar extent by serum samples from COVID‐19 patients [8, 10, 12, 29]. The detection of IFN‐ω mRNA in oropharyngeal swabs of COVID‐19 patients [30] and detection of NAB against IFN‐ω in severe COVID‐19 are indirect evidence for a possible role of IFN‐ω in the pathogenesis of SARS‐CoV‐2 infection.

The biological significance of anti‐IFN‐I NAB is not well known. In line with previous studies [31, 32, 33], we found lower levels of blood IFN genes in hospitalized COVID‐19 patients compared to matched healthy controls. The mechanisms of impaired IFN‐I production in these conditions are largely unknown and can be related to the host (e.g., aging [34], pre‐existing comorbidities [35], and genetic defects [7, 36]) but also SARS‐CoV‐2‐specific mechanisms (e.g., viral immune escape [1]). Here, we also demonstrated that expression levels in peripheral blood mononuclear cells (PBMC) of surrogate markers of IFN bioactivity, ISG15 and ISG56, were reduced in COVID‐19 patients with circulating NAB against IFN‐I. Furthermore, a negative correlation was found between NAB titer against IFN‐α2 subtype and ISG15 transcript levels. These observations confirm the results of previous investigations, in which depressed levels of ISGs were seen in COVID‐19 patients, in which NAB developed [11]. Our longitudinal analysis showed that in the subgroup of critically ill COVID‐19 patients who tested positive for auto‐Abs neutralizing IFN‐α subtypes and IFN‐ω, the persistence of high NAB titers was correlated with lack of expression of ISGs after a median time of 15 days from the start of their hospital admission. By contrast, the impact of NAB against IFN‐α on IFN‐I transcription in PBMC was less pronounced, consistent with the known action of NAB to block interaction between IFN and its receptor [16, 37].

A strength of our study was the ability to perform a comprehensive analysis of both BAB and NAB to IFN‐I in a large number of COVID‐19 patients including a detailed assessment of antibody specificity and influence of auto‐IFN antibodies on biochemical and hematological parameters associated with high risks for severe COVID‐19. Some limitations should be discussed. First, we did not characterize auto‐Abs to IFN‐I in samples before COVID‐19. Second, we did not evaluate auto‐Abs to IFN‐I in patients not hospitalized for SARS‐CoV‐2 infection. Further longitudinal studies with serial serum and respiratory samples from COVID‐19 patients, including less severely infected patients, are needed to better characterize the biological and clinical significance of auto‐Abs against IFN.

In conclusion, auto‐Abs able to neutralize multiple IFN‐α subtypes, and IFN‐ω can be found in hospitalized COVID‐19 patients, especially male patients. Moreover, NAB positive patients, but not those with auto‐Abs without anti‐IFN neutralizing activity, have raised levels of CRP, and significant alterations in total WBC, neutrophils, and platelets, suggesting that NAB status and the resulting impairment of IFN response are important pathogenic factors for COVID‐19 severity.

## Materials and methods

### Patients

Serum samples were collected at the time of admission from adult patients (n = 360) seen in the Division of Infectious Diseases, Hospital of Sapienza University of Rome, Italy with a clinical diagnosis of SARS‐CoV‐2 infection during the time period from March 2020 to April 2021. Paired respiratory (BAL or NPS) and serum samples were collected from 17 out of the 360 patients. A subset of patients (n = 7) had one or two follow‐up serum samples collected after hospitalization. Blood samples were obtained from gender‐ and age‐matched healthy controls (n = 19). The local ethics committee approved the study protocol (Sapienza University of Rome, University Hospital “Policlinico Umberto I”). All study participants gave written informed and patients’ data were anonymized.

### ELISA for quantitative detection of anti‐IFN‐α and anti‐IFN‐β BAB

Serum samples were screened for BAB against IFN‐α subtypes and IFN‐β using ELISA assays (anti‐IFN alpha Antibody Human ELISA Kit, Invitrogen, Thermo Fisher Scientific Inc. Vienna, Austria; Anti‐IFN beta Antibody Human ELISA Kit, Cloud‐Clone Corp. CCC, USA) according to the manufacturer provided protocol.

### Bioassay for detection of NAB against IFN‐α, ‐β, and ‐ω

Binding antibody positive serum samples were assayed for NAB to IFN‐α2 subtype (Intron; Schering‐Plough, Kenilworth, New Jersey, USA), IFN‐α subtypes contained in the natural IFN‐α preparation (IFN‐αn1, Wellferon, Glaxo Wellcome, Beckenham, UK), IFN‐β (Rebif, Serono, Geneva, Switzerland), and IFN‐ω (PBL Interferon Source, Piscataway, USA) in a bioassay based on IFN‐induced inhibition of encephalomyocarditis virus cytopathic effect on human lung carcinoma epithelial cells (A549) [9]. Briefly, twofold serial dilutions (starting from 1:10) of heat‐inactivated serum were incubated at 37°C with 20 IU/mL of the different IFN‐I preparations. After 1 h, the mixtures were added to duplicate monolayers of A549 cells (3 × 10^4^ cell/well) in 96‐well microtiter plates. After 24 h, the cells were challenged with encephalomyocarditis virus (MOI = 0.05 TCID_50_/cell) and incubated at 37°C for 24 h. Controls included a titration of each IFN‐I preparation. Antiviral activity and its neutralization were assessed based on virus‐induced cytopathic effect. Cells were stained with crystal violet and the dye taken up by the cells was measured in a spectrophotometer at 570 nm. Titers were calculated using the Kawade's method, and the titers were expressed in TRU/mL, where one TRU was the serum dilution able to reduce IFN titer from 10 to 1 IU/mL [38].

### TaqMan‐based real‐time RT‐PCR assays for IFN‐I‐related gene expression

The mRNAs levels of IFN‐I‐related genes were measured in PBMC and respiratory samples by quantitative RT/real time PCR assay as previously reported [39, 40]. The following primers and probes targeting IFN‐I genes were purchased from Integrated DNA Technologies (Coralville, IA, USA): IFN‐α2 (Hs.PT.58.24294810.g), IFN‐β1 (Hs.PT.58.39481063.g), IFNW1 (Hs.PT.5820160308.g). Primers and probes sequences for ISG15 and ISG56 were previously reported [39, 40]. The housekeeping gene β‐glucuronidase was used as an internal control to normalize the amount of total RNA of target genes. All real time PCR reactions were performed in duplicate. Gene expression values were calculated using the threshold cycle relative quantification (the 2^−Δ^ *^Ct^* method).

### Statistical analysis

Differences in frequencies of NAB and BAB between patient groups were determined using Yates Chi square or Fisher's exact tests. Differences in biochemical and hematological parameters between BAB/NAB negative or positive patients, in blood IFN‐I transcript levels between healthy donors, NAB positive and BAB negative patients, and in mRNA levels of IFN genes in respiratory samples of NAB positive and negative patients were determined using the Mann–Whitney test. For longitudinal analysis, the Wilcoxon signed‐rank test for paired samples was used to evaluate differences in IFN genes levels. Spearman's rho coefficient was calculated to assess the correlation between levels of NAB and IFN‐α2 subtype and the natural IFN‐α preparation and between anti‐IFN‐α2 NAB titer and ISG15 mRNA levels. Statistical analyses were carried out using SPSS software, version 26.00 (IBM).

## Conflict of interest

The authors declare no commercial or financial conflict of interest.

## Author contributions

FF, methodology, investigation, data curation, formal analysis. MS, methodology, investigation, data curation, formal analysis. LS, methodology, investigation, formal analysis. LG, conceptualization, validation, funding acquisition. OG, investigation. AC, investigation. AP, conceptualization, supervision. AA, conceptualization. CMM, conceptualization. GdE, conceptualization, clinical analysis. RPV, conceptualization, writing, supervision. GA, funding acquisition, conceptualization. CS, conceptualization, funding acquisition, methodology, resources, investigation, supervision, writing.

### Peer review

The peer review history for this article is available at https://publons.com/publon/10.1002/eji.202249824

**Abbreviations**
auto‐Abs: autoantibodies
BAB: binding antibodies
BAL: bronchoalveolar lavage
COVID‐19: coronavirus disease 2019
CRP: C‐reactive protein
ICU: intensive care unit
ISGs: IFN‐stimulated genes
NAB: neutralizing antibodies
NLR: neutrophils to lymphocytes ratio
PLR: platelets to lymphocytes ratio
WBC: white blood cells

## Supporting information

Supporting information

## Data Availability

The data that support the findings of this study are available from the corresponding author upon reasonable request.
