# Supplementary material for: Anti‐IFN‐α/‐ω neutralizing antibodies from COVID‐19 patients correlate with downregulation of IFN response and laboratory biomarkers of disease severity
Source: Eur J Immunol. 2022 Apr 28;52(7):1120–8. doi: 10.1002/eji.202249824 (PMC9087404; doi:10.1002/eji.202249824)
Supplement: Supplementary file 1 — Supporting information [file EJI-52-1120-s001.pdf]

Supplementary Table 1. Number of COVID-19 patients with anti-IFN-ω neutralizing antibodies (NAB, %) stratified according to anti-IFN-α2 NAB titer, gender, and clinical parameters

Patients stratified according to anti-IFN-α2 NAB titer (%) (n=13)	Number of anti-IFN-ω NAB patients (%)	Number of anti-IFN-ω NAB male patients (%) *	Number of anti-IFN-ω NAB patients admitted to ICU (%) **	Number of anti-IFN-ω NAB patients with fatal outcome (%) ***
3/13 (23) (Low: < 1000, range 500 – 13 TRU/ml)	1/3 (33.3)	1/1 (100)	0/1 (0)	0/1 (0)
4/13 (30.8) (Intermediate: ≥ 1000, range 8533 – 5688 TRU/ml)	2/4 (50)	1/2 (50)	1/2 (50)	1/2 (50)
6/13 (46.1) (High: ≥ 10.000, range 34133 – 10666 TRU/ml)	6/6 (100)	6/6 (100)	6/6 (100)	6/6 (100)

Data are expressed as proportion of COVID-19 patients with anti-IFN-α2 or IFN-ω NAB (%). Statistical analysis was performed using Fisher's exact test or Yates Chi-square. *Male sex association: **p=0.0034** [anti-IFN-ω NAB male patients (8/9) vs anti-IFN-ω NAB female patient (1/9)]; **ICU admission: **p<0.0001** [anti-IFN-I BAB negative patients (42/299) vs anti-IFN-ω NAB positive patients (7/9)]; ***Death rate: **p<0.0001** [anti-IFN-I BAB negative patients (32/299) vs anti-IFN-ω NAB positive patients (7/9)].

Supplementary Table 2. Anti-IFN-I neutralizing antibodies (NAB) in samples collected from the respiratory tract of COVID-19 patients

Patients No.	Sex	Age (years)	Respiratory sample	Days of hospitalization	Clinical characteristics	NAB to IFN-α2 (TRU/ml, serum samples)	NAB to IFN-α2 (TRU/ml, respiratory samples)	NAB to IFN-αn1* (TRU/ml, respiratory samples)	NAB to IFN-ω (TRU/ml, respiratory samples)	NAB to IFN-β (TRU/ml, respiratory samples)
14	M	79	BAL	72	ICU	<10	<10	<10	<10	<10
15	M	69	BAL	15	ICU	<10	<10	<10	<10	<10
16	F	76	BAL	12	ICU, dead	<10	<10	<10	<10	<10
17	M	73	BAL	1	ICU, dead	<10	<10	<10	<10	<10
18	F	63	BAL	NA	ICU, dead	<10	<10	<10	<10	<10
19	M	38	BAL	65	ICU, ECMO, dead	<10	<10	<10	<10	<10
20	M	67	BAL	60	ICU, dead	<10	<10	<10	<10	<10
21	M	80	BAL	91	VMK, CPAP	<10	<10	<10	<10	<10

7*	M	70	BAL	43	ICU, ECMO, diabetes, hypertension, IHD, DVT, dead	**17066**	**20**	**15**	<10	<10
22	F	80	BAL	130	ICU	<10	**15**	**10**	<10	<10
23	F	73	BAL	10	ICU, dead	<10	**10**	<10	<10	<10
24	F	67	NPS	NA	Oxygen support	<10	<10	<10	<10	<10
25	F	29	NPS	NA	No oxygen support	<10	<10	<10	<10	<10
26	F	62	NPS	12	Oxygen support	<10	<10	<10	<10	<10
27	M	54	NPS	14	NA	<10	<10	<10	<10	<10
28	F	62	NPS	NA	NA	<10	<10	<10	<10	<10
29	M	52	NPS	20	NA	<10	<10	<10	<10	<10

Clinical characteristics were available of 14 out of 17 COVID-19 patients. NAB positive samples are in bold. IFN-αn1* = is referred to IFN-α subtypes contained in the natural IFN-α preparation. ICU= intensive care unit; ECMO= extracorporeal membrane oxygenation; VMK= venturi mask; CPAP= continuous positive airway pressure; IHD= ischemic heart disease; DVT= deep vein thrombosis; BAL=bronchoalveolar lavage; NPS=nasopharyngeal swab; NA=not available.

Supplementary Figure 1

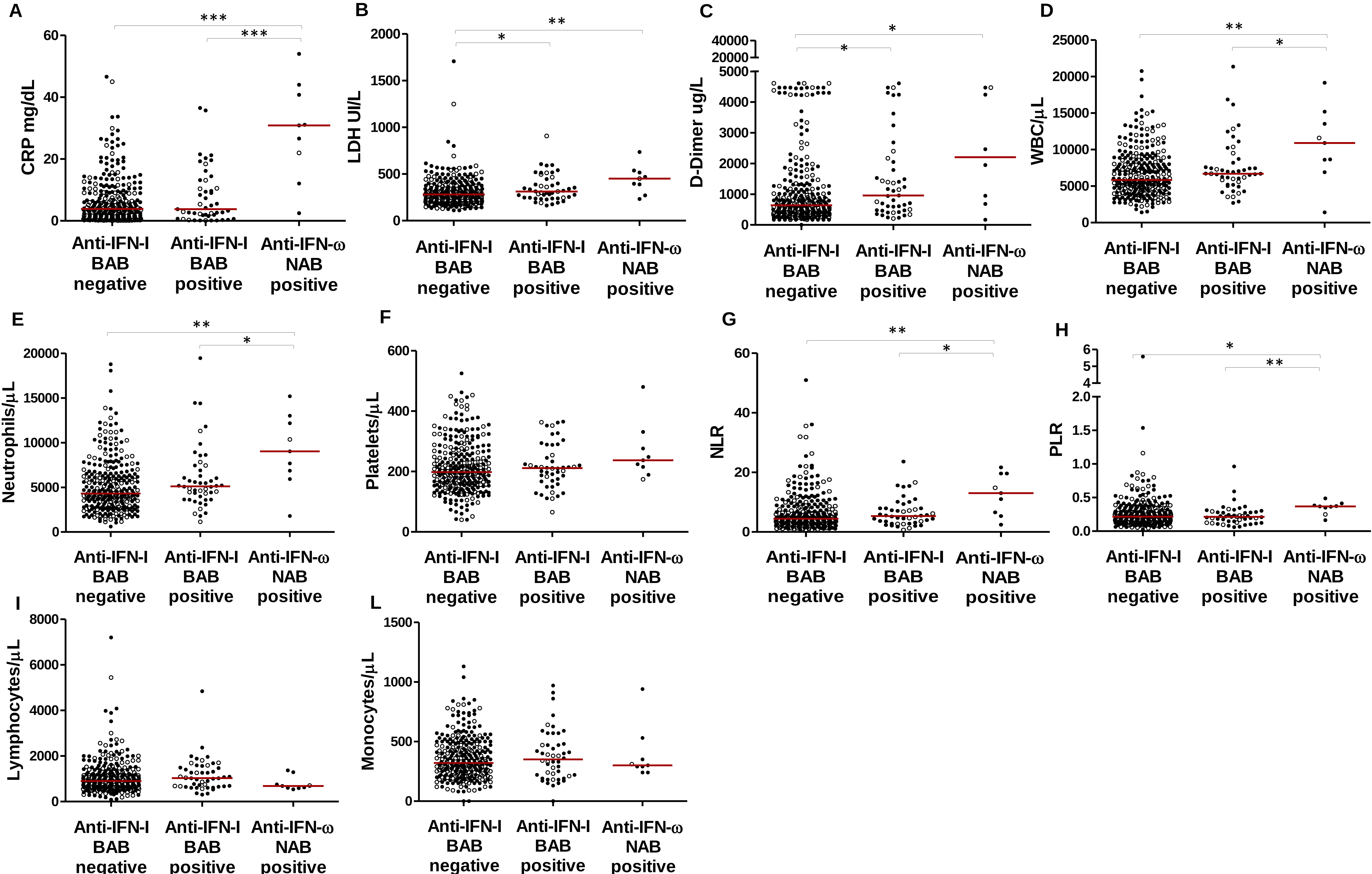

Supplementary Figure 1. Anti-IFN-ω NAB were associated with laboratory biomarkers predictive for COVID-19 outcome. Biochemical and hematological parameters measured in SARS-CoV-2 infected patients, stratified by auto-antibody status as anti-IFN-I BAB negative patients (n=299), anti-IFN-α/β BAB positive patients without NAB to IFN-α subtypes, IFN-β and IFN-ω (n=48), and anti-IFN-ω NAB positive patients (n=9). CRP= C-reactive protein (Panel A); LDH= lactate dehydrogenase (Panel B); D-Dimer (Panel C); WBC= white blood cells (Panel D); neutrophils (Panel E); platelets (Panel F); NLR= neutrophils to lymphocytes ratio (Panel G); PLR=platelets to lymphocytes ratio (Panel H); lymphocytes (Panel I); monocytes (Panel L); Median values of biochemical and hematological parameters are reported, for each group of study, with a red horizontal line. Female patients are represented with open circle symbols while male patients with close circle symbols. *$p<0.05$; **$p<0.01$; ***$p\leq 0.001$. Values of biochemical and hematological parameters were compared by Mann-Whitney test.

Supplementary Figure 2

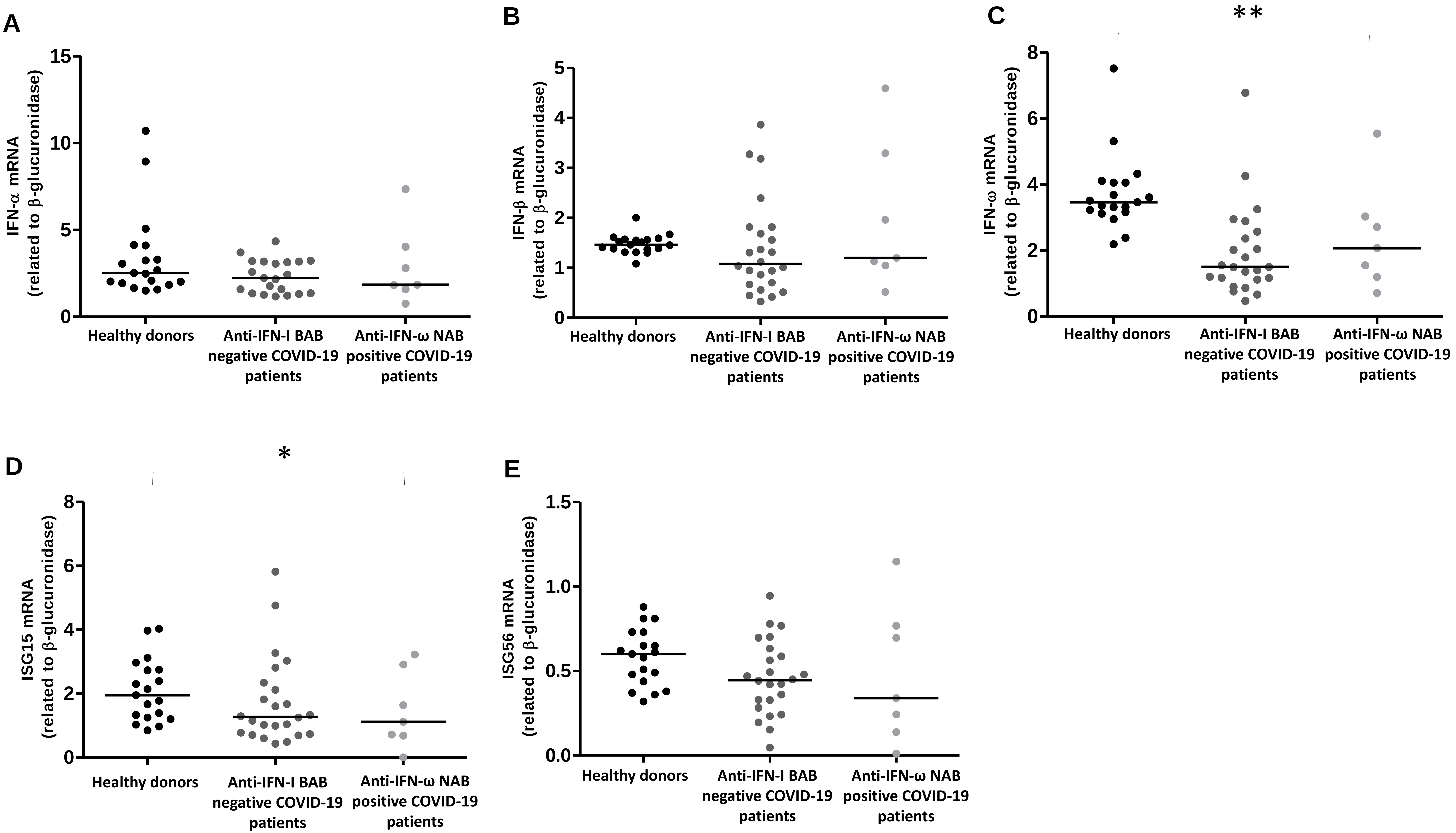

Supplementary Figure 2. Expression levels of IFN-I and IFN stimulated genes (ISGs) in anti-IFN-ω NAB positive COVID-19 patients. Panels A-E represent expression levels of genes encoding IFN-α (Panel A), IFN-β (Panel B), IFN-ω (Panel C), ISG15 (Panel D) and ISG56 (Panel E) in PBMC collected from healthy donors (n=19), anti-IFN-I BAB negative COVID-19 patients (n=24), and those who developed anti-IFN-ω NAB (n=7). Gene expression data were available for 7 out of 9 patients with NAB to IFN-ω. Statistical analysis of transcript levels of IFNs and ISGs genes related to β-glucuronidase ($2^{-\Delta Ct}$) method was carried out using Mann-Whitney test. Median values of gene expression levels are reported, for each group of study, with a black horizontal line. *p <0.01; **p <0.001.

Supplementary Figure 3

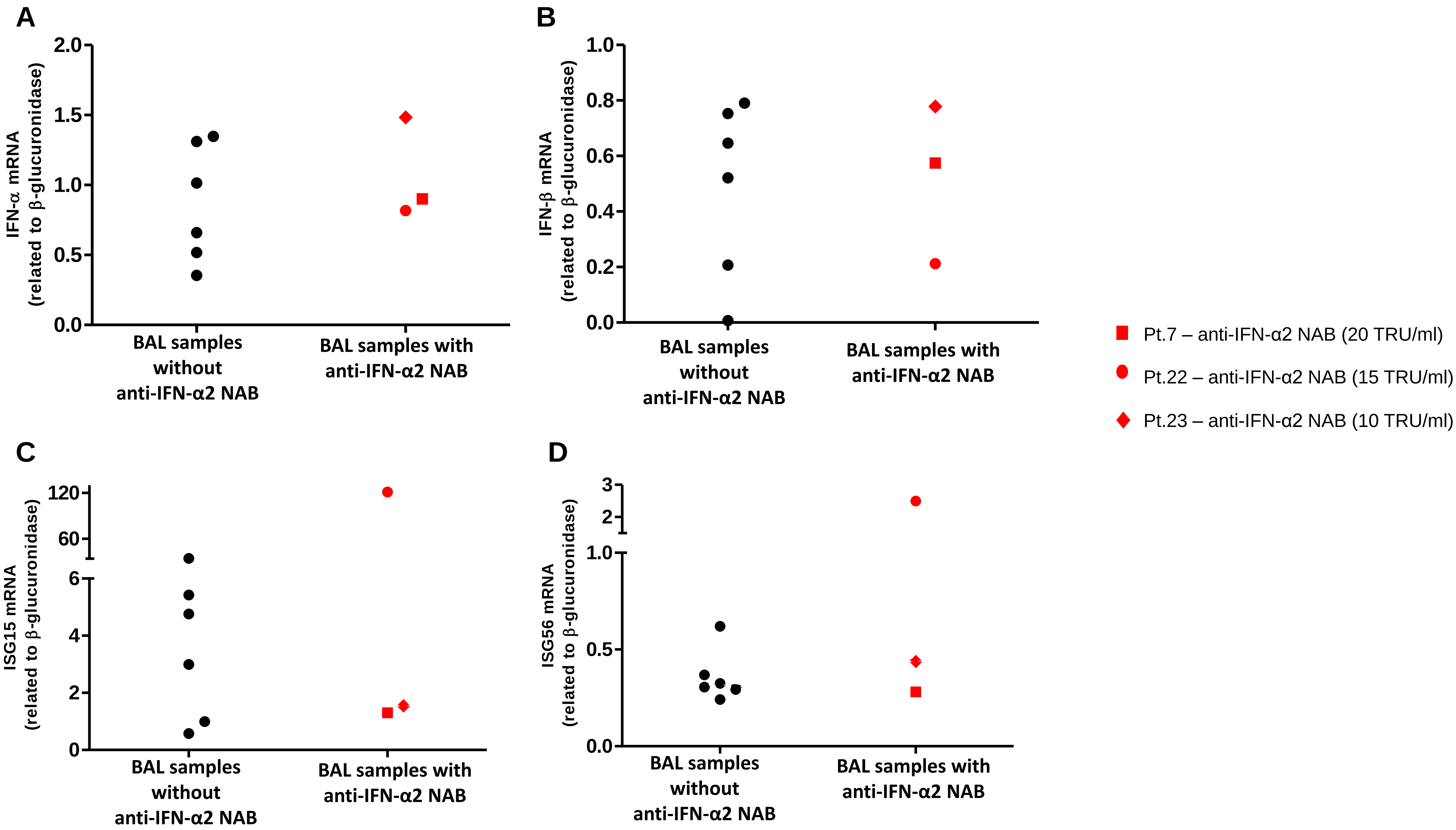

Supplementary Figure 3. Expression levels of IFN-α/β and IFN stimulated genes in respiratory samples of anti-IFN-α NAB positive COVID-19 patients. Expression levels of genes encoding IFN-α (Panel A), IFN-β (Panel B), ISG15 (Panel C) and ISG56 (Panel D) in bronchoalveolar lavage (BAL) collected from COVID-19 patients with (n=3, Pt 7, Pt 22 and Pt 23) or without anti-IFN-α2 NAB (n=6). For each patient (Pt 7, Pt 22 and Pt 23) is indicated the NAB titer against IFN-α2 subtype. Distinct red symbols (square, circle and rhombus) represent COVID-19 patients (n=3) with anti-IFN-α2 NAB in BAL samples. IFN-β data were available for 5 out of 6 BAL samples without anti-IFN-α2 NAB.
